# Investigating the validation of the Chinese Mandarin version of the Social Responsiveness Scale in a Mainland China child population

**DOI:** 10.1186/s12888-016-1185-y

**Published:** 2017-02-06

**Authors:** Chao-Qun Cen, Ya-Yong Liang, Qiu-Ru Chen, Kai-Yun Chen, Hong-Zhu Deng, Bi-Yuan Chen, Xiao-Bing Zou

**Affiliations:** 1 Child Developmental-Behavioral Center, The Third Affiliated Hospital of Sun Yat-sen University, No. 600, Tianhe Rd., Guangzhou, 510630 China; 2 Department of Pediatrics, Huizhou First Hospital, NO.20, South Sanxin Rd., Huizhou, 516000 China

**Keywords:** Reliability, Validity, Social Responsiveness Scale, Chinese version, Autism spectrum disorder

## Abstract

**Background:**

Researchers from several different countries have found the Social Responsiveness Scale (SRS) to have good psychometric properties. However, to our knowledge, no studies on this subject have been reported in Mainland China. In this study, we investigated the psychometric properties of the Chinese Mandarin version of the SRS when used in Mainland China.

**Methods:**

The reliability and validity of the parent-report SRS in a sample of 749 children of 4- to 14-year-olds: 411 typically developing and 338 clinical participants (202 with autism spectrum disorder (ASD)) were examined.

**Results:**

Internal consistency for total scale (0.871–0.922), test–retest reliability (0.81–0.94), and convergent validity with the Autism Behavior Checklist (ABC) (0.302–0.647) were satisfactory. The SRS total score discriminated between the ASD and other developmental disorders. Receiver operating characteristic (ROC) analyses revealed that the SRS was predicted to accurately classify 69.2–97.2% of youth ASD. Exploratory factor analysis (EFA) supported a single-factor solution for the ASD subsample. Confirmatory factor analysis (CFA) did not confirm the theoretical construct of five factors model with inadequate fit in the ASD subsample.

**Conclusions:**

Overall, our findings supported the reliability and validity of the parent-report SRS as one ASD screening instrument. In addition, we also suggest that the use of separate cut-offs for screening purposes (optimizing sensitivity) vs. clinical confirmation (optimizing specificity) should be considered.

## Background

The Diagnostic and Statistical Manual of Mental Disorders (DSM-IV) [1] defines Autism Spectrum Disorders (ASD) as a group of developmental disabilities characterized by impairments in social interaction and communication, and by restricted, repetitive, and stereotyped patterns of behavior. The most common categories include Autism, Asperger syndrome (AS), and Pervasive development disorder-not otherwise specified (PDD-NOS), all currently conceptualized to lie on a continuum of autism-specific traits. The above definition, which contains only one diagnosis of ASD with varying degrees of severity, has been used by the DSM-V [2].

The prevalence of ASD has been reported to be constantly increasing. A 2012 review of global prevalence estimates of ASD found a median of 62 cases per 10,000 people [3]. The prevalence estimates are presently up to 1 in 68 children under 8 years of age in the USA [4]. In China, there has been a lack of large-scale epidemiological investigations on ASD. Sun. et al. [5] performed a systematic review and meta-analysis of relevant studies (from 1987 to 2011) from Mainland China, Hong Kong and Taiwan. The results indicated the prevalence of childhood autism in mainland China was 11.8/10,000, while pooled prevalence of ASD in all three areas was 26.6/10,000, which is significantly lower than that reported by above mentioned studies. Based on assessing the research methodologies of different prevalence estimates studies, the authors concluded that there was a potential under-diagnosis and under-detection of ASD in mainland China, Hong Kong and Taiwan. Considering that early detection of ASD can lead to treatments and support which improve functioning and quality of life, there is an urgent need for an efficient screening or identifying tool for ASD to facilitate the diagnose and further intervention in China.

The Autism Diagnostic Interview-Revised (ADI-R) [6] and the Autism Diagnostic Observation Schedule (ADOS) [7] are considered to be the ‘gold standard’ in diagnostic evaluations for autism, particularly when combined with clinical judgment [8]. However, the autism research and clinical communities today face a global imbalance in our knowledge of autism and corresponding disparities in access to autism screening, diagnosis, and treatment exhibit globally [9]. Both ADI-R and ADOS are costly, require extensive training, and are lengthy to administer, which limits their feasibility in clinical settings [10], especially in low resource countries [9]. Consequently, ADI-R and ADOS cannot be administered as a routine part of the ASD evaluations in the clinical settings in China, due the lack of resources available for related assessment, diagnosis and intervention, and as well as restricted cover range of medical insurance. As already mentioned, rapid detection of ASD permits early diagnosis and treatment, which in turn increases the prognosis reliability for the patient [11, 12]. Therefore, time and cost effective measures which can identify potential at-risk cases for further assessments are urgent for the purpose of clinical and research settings in China.

Due to their easy applications and low cost, the Childhood Autism Rating Scale (CARS) [13] and the Autism Behavior Checklist (ABC) [14] have been frequently used in China as part of the diagnostic process in research and in clinical practice. The CARS is used to observe and subjectively rate fifteen items, scored from one to four for various criteria, ranging from normal to severe. The ABC consists of 57 items which are a list of atypical behaviors characteristic of the pathology and it is designed for the triage of children suspected of having autism. However, both above-mentioned assessing instruments may exhibit some of the following problems. First, proportion of the items which are associated with the repetitive and stereotyped behaviors and interests is too large in the ABC; Second, descriptions of the items in both assessments are excessively general and lack of specific behavioral descriptions, which may be considered to be ambiguous, giving rise to different interpretations; Third, most of the items in both assessments are about descriptions of severe autistic symptoms and very little is related with normal behaviors, which, consequently, would be difficult to reflect the concept of spectrum in ASD which ranging from mild to severe.

The Social Responsiveness Scale (SRS) [15] is the first widely used quantitative parent/ teacher-report measure of autistic traits for use in the general population, as well as in educational and clinical settings [10]. SRS was developed in the USA, along with the general population norms reported for USA respondents by the authors [15]. It has been used in a number of other countries or regions, including the UK [16], Germany [17, 18], Mexico [19], Canada [20], Netherlands [21], Australia [22], and Taiwan [23, 24]. It has been employed in a variety of ways: as a measure of the severity or quantities of social impairment in clinical sample including ASD, a measure of the quantities of ASD-like traits in non-clinical or general sample (a general population screening tool), and for genetic [21, 25–27] and intervention [28] evaluation studies. Preceding studies by the developer and his colleagues had investigated and demonstrated good psychometric properties of the SRS [29, 30]. The USA general population norms were reported by the authors based on this scale in 2005. A summary of some of the more recently published study outcomes on the psychometric properties of the SRS version for 4–18 years old was presented in Table 1.

**Table 1:** Reliability and validity studies on the SRS version for 4–18 years old

Authors and year	Sample	Rating way	Area and main findings
1. Constantino et al. [2007] [40]	PDD 271, Siblings of PDD 254, non-PDD clinical 52	Teacher/parent	Teacher/parent correlation: 0.72. Correlations with ADI-R: 0.15–0.43(teacher rating), 0.58–0.39(parent rating). Correlations with ADOS: 0.26–0.40(teacher rating), 0.31–0.36(parent rating). ROC curve of the PDD vs. non-PDD clinical and TD combined: AUC 0.95, T-score cut-off 60 resulted sensitivity 0.75 and specificity 0.96(both a parent and a teacher rating), specificity 84%(parent only), and specificity 90%(teacher only).
2. Bölte et al. [2008] [17]	TD 838, clinical527 (ASD 160, ADHD 134, other 233)	Parent	Internal consistency: 0.91–0.97. Test–retest reliability: 0.84–0.97. Interrater reliability: 0.76 and 0.95. Correlation with ADOS, ADI-R and SCQ: 0.35–0.58. Discriminant validity: total score discriminates ASD from other clinical conditions. ROC analysis (ASD vs. other clinical): AUC 0.83, total score 85 had sensitivity 0.73 and specificity 0.81 Factorial validity: One-factor solution for normative and clinical subsamples.
3. Murray MJ. et al. [2011] [37]	29 suspected ASD	Parent	Agreement between ADI-R: 89.7%, kappa of 0.51. Correlation with CASD: 0.40 Correlation with ADI-R : non-significant
4. Bölte et al. [2011] [18]	ASD 148, non-ASD clinical 255, TD 77	Parent	Internal consistency: 0.96 (ASD), 0.94 (non-ASD clinical) and 0.91 (TD). Correlations with the ADI-R, ADOS, SCQ and SCDC were: 0.31–0.45, 0.32–0.35, 0.50, and 0.49. ROC analysis of ASD vs. TD: AUC 0.98, sensitivity 0. 80 and specificity 1.0 (total score 75); sensitivity 0.74 and specificity 1.0 (total score 85). ROC analysis of ASD vs. non-ASD clinical: AUC 0.81, sensitivity 0. 80 and specificity 0.69 (total score 75); sensitivity 0.74 and specificity 0.79 (total score 85). ROC analysis of ASD vs. ADHD: AUC 0.86, sensitivity 0. 80 and specificity 0.78 (total score 75); sensitivity 0.74 and specificity 0.83 (total score 85).
5. Aldridge FJ. et al. [2012] [22]	48 suspected ASD	Teacher/parent	Diagnostic Sensitivity: 91% (parent report), 84% (teacher report) Diagnostic specificity: 8% (parent report), 41% (teacher report).
6. Schanding GT. et al. [2012] [38]	ASD 1663, siblings of ASD 1712	Teacher/parent	Correlation with SCQ, ADOS, and ADI-R: 0.73, 0.35- 0.38, 0.08 - 0.25 (teacher rating). Correlation with SCQ, ADOS, and ADI-R: 0.59, 0.12 - 0.16, 0.25 - 0.39 (parent rating). ROC analysis: AUC 0.935 (teacher rating) 0.988 (parent rating), total score 60 had sensitivity 0.694 and specificity 0.953 (teacher rating), total score 75 had sensitivity 0.800 and specificity 0.994 (parent rating).
7. Wigham S.et al. [2012] [16]	52with and 414 without special needs	Parent	Internal reliability: 0.92 (total scale), 0.47-0.83 (subscales). Correlation with RBQ2 and SDQ: 0.445 and 0.704. Discriminant validity: total score discriminated children with ‘special needs’ and the ‘no special needs’ group. Principal components analysis: a single factor structure
8. Wang J. et al. [2012] [23]	TD 140, clinical 167 (ASD, ADHD and other).	Parent	Internal consistency of total scale: 0.85 (TD), 0.87 (ASD), 0.92–0.94 (other clinical). Internal consistency of subscale scale: 0.40–0.71 (TD), 0.36–0.83 (ASD), 0.43–0.90 (other clinical). ROC analysis: cut-off 85 showed AUC 0.88, sensitivity0.66, specificity0.89. Suggested optimal cut-off for screening 65 with sensitivity 0.94 and specificity 0.70; Suggested optimal cut-off for clinical classification 87 with sensitivity 0.66 and specificity 0.90.
9. Fombonne E. et al. [2012] [19]	ASD 200, TD 363	Teacher/parent	Internal consistency for parent rating: 0.97 (total scale), 0.73–0.93 (subscale). Internal consistency for parent rating: 0.97 (total scale), 0.79–0.93 (subscale). Parent-teacher correlations: 0.49 (total scale):0.22–0.50 (subscale). ROC analysis: AUC 0.962, optimal cut-off was 60 presented sensitivity 92.5% and specificity 92.6% (parent rating). ROC analysis: AUC0.960, optimal cut-off was 59 presented sensitivity 93.6% and specificity 84.3% (teacher rating). ROC analysis: AUC 0.984, optimal cut-off was 61 presented sensitivity 97.9% and specificity 88.1% (average of parent and teacher scores). ROC analysis: AUC0.970, optimal cut-off was 87 presented sensitivity 86.4% and specificity 94.0% (highest value of parent and teacher scores).
10. Gau F. et al. [2013] [24]	TD 1419, ASD 401	Parent	Factor analysis: exploratory factor analysis yielded a 4-factor structure which was validated by confirmatory factor analysis with an adequate fit after excluding five items with low correlation coefficients. Test-retest reliability: 0.751–0.852. Internal consistency: 0.944–0.947. Correlations with SCQ: 0.609–0.865. Discriminant validity: ASD significantly higher than TD on total scale and subscales.
11. Duku E. et al. [2013] [20]	ASD 205	Parent	Internal consistency: 0.93 (total scale), 0.60–0.85 (subscale). Confirmatory factor analysis: did not fit well and were not unidimensional. Rasch analyses: showed a 30-item subset met criteria of unidimensionality. Correlations between 30-item subset and 65-item total raw score: 0.94. (30-item subset) Correlations with CBCL, RBS-R: 0.65–0.68.
12. Pearl AM. et al. [2013] [52]	ASD 26; TD24	Parent	Mother-father reliability: 0.92 (total scale), 0.80–0.93 (subscales).
13. Cholemkery H. et al. [2014] [39]	High-functioned ASD 55, ODD/CD 55, TD 55	Parent	ROC analysis of ASD vs. TD: AUC1.0, cut-off 43 showed sensitivity 0.98 and specificity 0.95. ROC analysis of ASD vs. ODD/CD: AUC 0.82, total score 80 showed sensitivity 0.76 and specificity 0.82. Combination with three other parent-rated questionnaires improved validity to differentiate ASD and ODD/CD. Correlations with SCQ, ADI-R, CBCL: 0.55, 0.33 and 0.78.

Note: *ADI-R* autism diagnostic interview-revised, *ADOS* autism diagnostic observation schedule, ROC receiver operating characteristics, *AUC* area under the curve, *PDD* pervasive developmental disorder, *TD* typical developmental, *ADHD* attention deficit hyperactivity disorder, *CASD* checklist for autism spectrum disorder, *SCQ* social communication questionnaire, *RBQ2* repetitive behaviors questionnaire 2, *SDQ* strengths and difficulties questionnaire, *CBCL* child behavior checklist, *DD* unspecific developmental disorders, *RBS-R* repetitive behavior scale-revised, *ODD* oppositional defiant disorder, *CD* conduct disorder, *SRS* social responsiveness scale

While there have been studies regarding the investigation of the utilities of Chinese Mandarin version of SRS (Chinese SRS) in Taiwan, none have been reported yet in Mainland China. Although Mainland China and Taiwan are both Chinese-speaking areas, they are considered to have cultural differences. Furthermore, Mainland China is the largest or broadest Chinese-speaking region; despite the shared language between the Mainland China and Taiwan, it is necessary to carry out separate investigations on the usefulness of Chinese SRS in Mainland China. Therefore, the aim of the present study was to examine the psychometric properties of the Chinese SRS when used in a sample from Mainland China.

## Method

### Participants

Participants of our study included clinical group and typically developing (TD) group, children aged 4–14 years. The clinical study group was composed of ASD, Attention Deficit Hyperactivity Disorder (ADHD), and Mental Retardation (MR) subgroups which were referred for developmental evaluation at the Child Developmental & Behavioral Division of the Third Affiliated Hospital of Sun Yat-sen University in Guangzhou, China, from April 2012 to July 2013. This hospital is a large-scale medical center in Mainland China. It’s Child Developmental and Behavioral Division is well known in Mainland China for specializing in the assessment, diagnosis and intervention related services for developmental disorders and conditions. Parents of a total of 338 children in the clinical group were consented and participated in the present study. The ASD subgroup (*n* = 202), included 98 individuals with autism, 63 with AS and 41 with PDD-NOS. The ADHD subgroup (*n* = 73) consisted of three subtypes: predominantly inattentive, predominantly hyperactive-impulsive, and the combined. 63 individuals were included in the MR group. All clinical diagnoses were confirmed by two developmental and behavioral pediatricians with extensive clinical and research experience in the assessment and treatment of children and adolescents with such conditions. The diagnoses were established by integrating data from parental interviews, developmental history and medical records, with information provided by other Caregivers and teachers, and direct observation and interaction with the study participants after at least three assessment visits. All diagnoses fulfilled the corresponding criteria of DSM-IV.

The TD group consisted of 411 children aged 4–14 years, whose parents were consented and participated in the study. In December 2012, we randomly selected one local kindergarten and one local primary school to also participate in the study. Two classes from each grade level (grades small, middle and junior in kindergarten aged 4–6; grades 1–6 in primary school, aged 6–14) were randomly selected from each school. Following, 15 children from each selected kindergarten class (90 in total) and 30 to 35 children from each selected primary school class (400 in total) were randomly chosen. These children had not previously been reported having developmental or psychiatric conditions, which were determined by information provided by their teachers. 411 participants in total were included in the final research. The participation rate was 83.9%.

In the TD group, the majority of the fathers and mothers of the participants were college graduates (89.0 and 84.9%) and about ten percent or above were senior high school graduates (9.7 and 13.9%). In the clinical group, more than half of the fathers and mothers were college graduates (58.8 and 56.5%) and about one quarter were senior high school graduates (25.0 and 23.8%), respectively. In terms of economic status, about one quarter of the participating families were in good economic standing (28.2%) and one quarter, in middle to low economic standing (71.8%) in the clinical group, and 40.5 and 49.5% respectively in the TD group. In addition, most of the respondents in both TD group (75.3%) and in clinical group (81.8%) were mothers.

### Instruments

#### Social Responsiveness Scale (SRS)

The present study used the Chinese SRS version for children aged 4–18 years. The SRS [15] is a 65-item questionnaire. Each item is scored on a Likert scale ranging from 1 (not true) to 4 (almost always true). When completed, a total raw score (ranging from 0 to 195, with higher scores indicating increased social impairment) and five theoretical subscales scores (labeled social awareness, social cognition, social communication, social motivation, and autistic mannerisms) can be generated. SRS focuses on the child’s behavior during the past 6 months and can be completed in 15–20 min by a parent, a teacher, or another frequent Caregiver. The SRS can be used in the following areas: as an ASD screener, as an aid to clinical diagnosis, as a quantitative ASD trait measurement and as a measure to monitor the response to the intervention, because it is able to measure subtle changes in the severity of symptoms over time.

#### Autism Behavior Checklist (ABC)

In order to investigate the convergent validity of the SRS, Autism Behavior Checklist (ABC) [14] of Chinese version was used in the current study. ABC is one of the most commonly used ASD assessment instruments in Mainland China. Similar to the SRS, the ABC is an unstructured parent or Caregiver-reported questionnaire. The ABC addresses 57 atypical behaviors (scored 1 to 4) related to five areas: sensory behaviors, relating behaviors, body and object use behaviors, language behaviors, and social self-help behaviors. The psychometric properties of the ABC have been studied for some years, and it has been considered useful in the screening of the children suspected of having autism. The developer of this scale proposed the total raw score of 68 for the cut-off point [14]. The sensitivity ranged from 0.38 to 0.58, and the specificity ranged from 0.76 to 0.97 on the cut-off point 68. A systematic review of Brazilian studies related to psychometric properties of assessment instruments for autism spectrum disorder found that all studies aiming to validate instruments showed evidence of validity and sensitivity, and specificity values above 0.90 were observed in the ABC [31]. The data from a Mainland China children sample indicated that when 50 or 62 was used as the ABC cut-off point to screen autism from the normal people the sensitivity of this scale were 0.97 and 0.95 respectively and the specificity were both 1.00, and when cut-off point 50 or 62 was used to differentiate autism from mental retardation (MR) the sensitivity of ABC were 0.97 and 0.95 and the specificity were 0.85 and 0.90 respectively [32].

### Procedures

The parents of the participants of the clinical group (*n* = 338) completed the SRS, and the parents of the 82 participants from the ASD subgroup (*n* = 202) completed the ABC while in the waiting room of the Child Developmental and Behavioral Division for their first doctor-visit. Clinicians were blinded to the SRS scores and ABC scores of the children in the clinical study groups when making diagnoses.

The parents of the participants of the TD group (*n* = 411) completed the SRS at home and later returned them to their children’s teachers. Teachers at participating kindergarten sent the Caregivers of participants the questionnaire when they met the Caregivers of participants after the classes are over for that day. For the primary school sample, the questionnaire was delivered to the parents by the participants. The package included a description of the measuring purpose, an explanation on how to fill out the survey and the contact number for further inquiries regarding the first page of the questionnaire. The participation rate was 83.9%, that is, 411 respondents were included. We randomly selected 23 primary school participants (grades 1–4, aged 6–9, and 56.5% boys) from the TD group to fill out the same questionnaire again at a 2-week interval to examine test–retest reliability.

### Statistical analyses

Data analyses were performed using SPSS17.0 and AMOS 17.0 software. Raw SRS scores were used for all analyses. One-way Analysis of Variance (ANOVA) was carried out to compare mean age, SRS scores and IQ scores between four study groups (TD, ASD, ADHD, and MR), and Student-Newman-Keuls test (SNK) was performed for multiple comparison. Chi-square test was used to compare the sex distribution between the four study groups. T test was used to compare SRS scores between study groups (male vs. female, preschool age vs. school age). Spearman’s correlations were conducted to examine the correlation between total Raw SRS scores and age. Reliability was estimated by internal consistency (Cronbach’s alpha coefficient) of each subscale, and the total scale in TD and ASD sample, as well as test–retest reliability of the five subscales, and the SRS total score in TD sample using intra-class correlation. Criterion-related validity was analyzed in the ASD sample by using Spearman’s correlations to examine the relationships between SRS and the score of the ABC. Discriminant validity of each domain between different groups was explored by using ANOVA. The ability of the SRS to predict the diagnostic category for each of the cutoffs was examined by the receiver operating characteristic (ROC) analysis, using the area under the curve (AUC). The construct validity of the scale was explored using exploratory factor analysis (EFA) and confirmatory factor analysis (CFA). EFA was performed using principal factor and oblique promax rotation, which is an appropriate method for potentially correlated factors. CFA was performed by using the Structural Equation Model (SEM), and the model was fit to the data using the maximum likelihood (ML) estimation.

## Results

### Demographic and Wechsler IQ characteristics of the study groups

Seven hundred and forty nine cases were included in the final analyses. As shown in Table 2, male participants were more highly represented in the clinical subgroup than in the TD group (50.9% vs. 66.7–88.1%), especially in the ASD and ADHD subgroups, where 88.1 and 86.3% of participants, respectively, were male. The three clinical subgroups were significantly different from each other and also different from the TD group, except for the MR group in age. The children in the TD group did not complete the IQ test. Wechsler IQ level was evaluated in 56.9% of the children in the ASD subgroup and 100% in the ADHD and MR subgroups, respectively. The three clinical subgroups differed in mean VIQ, PIQ and FIQ, with the ADHD subgroup scoring the highest and the MR group the lowest (*P* < 0.001).

**Table 2:** Selected demographic characteristics, and Wechsler IQ by study groups

	TD	ASD	ADHD	MR	*F*/*χ* ^2^	*P*
n (749)	411	202	73	63		
Male: n (%)	209 (50.9)^a^	178 (88.1)^b^	63 (86.3)^b^	42 (66.7)^a^	99.021	<0.001
Age range	4–14	4–14	6–12	4–12		
Age (years): mean ± SD	7.43 ± 2.08^a^	6.37 ± 2.35^b^	8.41 ± 1.65^c^	7.66 ± 2.06^a^	20.611	<0.001
VIQ: mean ± SD	-	83.60 ± 28.05 ^a^	98.65 ± 15.77 ^b^	59.96 ± 15.09 ^c^	52.064	<0.001
PIQ: mean ± SD	-	84.88 ± 23.83 ^a^	92.72 ± 14.56 ^b^	64.96 ± 14.15 ^c^	37.044	<0.001
FIQ: mean ± SD	-	82.66 ± 26.65 ^a^	95.43 ± 14.57 ^b^	58.36 ± 14.79 ^c^	53.834	<0.001

Note: values in the same row with the different letter are statistically different at *P* < 0.01 (two-tailed)

### SRS total raw score means by gender and age

**Fig. 1:**
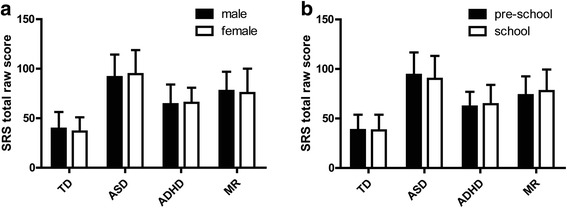
SRS total raw score means by gender (**a**) and age (**b**)

Table 3 and Fig. 1 showed the SRS total raw score means and standard deviations for male and female, as well as for preschool-aged and school-aged participants of the TD, ASD, ADHD and MR study groups. In all four groups, there were no significant differences between the female and male samples, and likewise, no significant differences between the preschool-aged and school-aged samples on the SRS total scores (*P* > 0.05). There is thus no evidence of systematic sex difference within this normative population, and norms do not require sex stratification.

**Table 3:** SRS total raw score means by gender and age

	Mean ± SD			
	Male	Female	Preschool-aged	School-aged
TD	39.38 ± 16.90	36.53 ± 14.40	38.32 ± 15.46	37.91 ± 15.85
ASD	91.59 ± 22.77	94.58 ± 24.17	94.11 ± 22.56	90.03 ± 23.13
ADHD	64.17 ± 19.94	65.60 ± 15.19	62.00 ± 14.93	64.47 ± 19.52
MR	77.57 ± 19.32	75.48 ± 24.60	73.62 ± 18.95	77.72 ± 21.66

### Correlations between SRS total raw score and age in various study groups

**Fig. 2:**
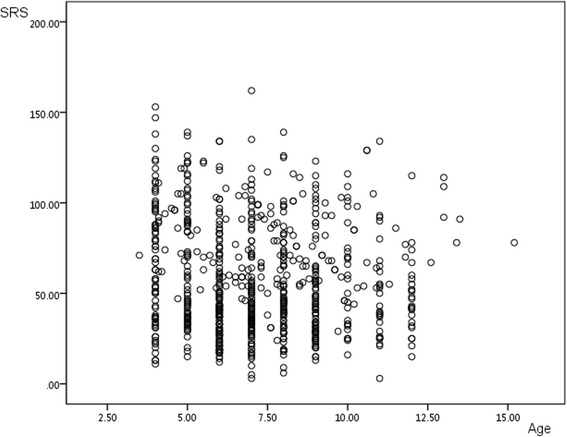
Scatter plots between SRS total raw score and age of the total sample

The correlation coefficient between the SRS total raw score and age in the TD, ASD, ADHD and MR groups were 0.087, −0.011, −0.062 and 0.080 respectively, which indicated non-significance (*P* > 0.05). The total sample (TD group and three clinical subgroups combined) resulted in a coefficient of −0.107 (*P* < 0.05), which was significant but much less than 0.40. Since scatter plots often show at a glance whether a relationship exists between two sets of data, we drew the scatter plots (Fig. 2) using the SRS total raw score and age as variables to investigate the possible relationship between the two in the total sample. As expected, there was no evidence of a linear trend in the scatter plots. Considering this together with the above results, one can suggest that there is no linear correlation of a significant level between the SRS total raw score and age.

### Test-retest reliability and internal consistency

Reliability data were summarized in Table 4. The test-retest reliability (2 weeks) in the TD group ranged from 0.81 to 0.94 for the subscale scores and was 0.96 for the SRS total raw score. The internal consistency (Cronbach’s alpha) of the total scale in the ASD and TD groups was around 0.90 for female, male and both samples. According to the subscales, the alpha values on “social awareness” (0.277 - 0.428) were always the lowest, while the alpha values on “social communication” (0.726–0.819) were, on the contrary, the largest in five subscales, independently of which group or sample of the above was examined. The alpha values on “social cognition” ranged from 0.556 to 0.698, and “social motivation” ranged from 0.613 to 0.709. Both “social communication” and “social mannerisms” had alpha values of more than 0.7.

**Table 4:** Test–retest reliability and internal consistency of the Chinese SRS

	Test–retest	Cronbach’s alpha					
		ASD			TD		
		Male	Female	All	Male	Female	All
Social awareness	0.81	0.428	0.277	0.410	0.320	0.283	0.316
Social cognition	0.87	0.698	0.620	0.689	0.603	0.556	0.580
Social communication	0.93	0.816	0.819	0.816	0.804	0.726	0.774
Social motivation	0.88	0.690	0.709	0.694	0.678	0.613	0.648
Autistic mannerisms	0.94	0.787	0.803	0.788	0.779	0.711	0.750
Total scale	0.96	0.917	0.922	0.917	0.906	0.871	0.892

### Concurrent validity

As shown in Table 5, all correlations with established autism scale were positive, significant (*P* < 0.001), and middle to good. In 82 respondents from the ASD group, the correlation of the SRS total raw score and the ABC total raw score was 0.634. The correlation between SRS total score and the ABC subscale score ranged from 0.509 to 0.647. The correlation between the ABC total score with the SRS subscale score ranged from 0.385 to 0.589. The SRS subscale score related to the ABC subscale score with the coefficient ranging from 0.302 to 0.635. When two instruments measure the same/similar attributes, correlation coefficients should be between 0.4 and 0.8 [33], thus it can be suggested that the concurrent validity of the SRS is acceptable.

**Table 5:** Pearson’s correlations between the subscales of the Chinese SRS and ABC

SRS	ABC					
	Sensory	Relating	Body and object use	Language	Social self-help	Total scale
Social awareness	0.370^*^	0.388^*^	0.322^*^	0.401^*^	0.302^*^	0.385^*^
Social cognition	0.494^*^	0.548^*^	0.491^*^	0.572^*^	0.458^*^	0.576^*^
Social communication	0.507^*^	0.635^*^	0.398^*^	0.590^*^	0.520^*^	0.589^*^
Social motivation	0.369^*^	0.625^*^	0.365^*^	0.425^*^	0.457^*^	0.524^*^
Autistic mannerisms	0.323^*^	0.364^*^	0.491^*^	0.411^*^	0.433^*^	0.449^*^
Total scale	0.516^*^	0.647^*^	0.509^*^	0.603^*^	0.553^*^	0.634^*^

* *P* <0.001

### Discriminant validity

**Fig. 3:**
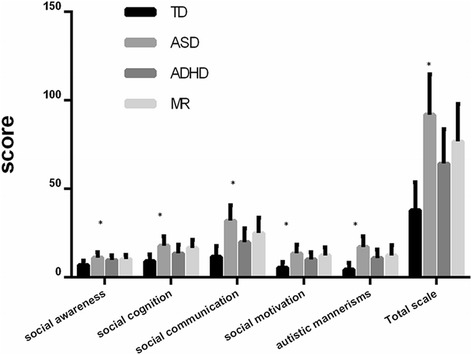
Analyses of discriminant validity of SRS. Note: * values in the same group are statistically different at *P* <0.001 (F-test two-tailed)

To test the discriminative validation of the SRS, we drew comparisons between the results from different study groups (i.e., TD, ASD, ADHD and MR), as shown in Table 6 and Fig. 3. As expected, we observed the highest SRS score means in children with ASD, followed by the other two clinical subgroups, while the TD group scored the lowest. The ASD subgroup clearly demonstrated higher scores for each subscale and the total scale than the TD group (*P* < 0.001). Furthermore, the ASD subgroup was distinguished from other two non-ASD clinical subgroups by a significant higher score means on the SRS total scale and five subscales (*P* < 0.001).

**Table 6:** Analyses of discriminant validity of SRS

	TD	ASD	ADHD	MR	*F*	P
Social awareness	7.03 ± 2.56^a^	11.21 ± 2.92^b^	9.79 ± 2.64^c^	10.27 ± 2.44^c^	125.199	<0.001
Social cognition	9.23 ± 3.86^a^	17.93 ± 5.20^b^	13.58 ± 4.89^c^	16.73 ± 4.59^d^	196.56	<0.001
Social communication	11.70 ± 6.08^a^	32.05 ± 8.66^b^	19.88 ± 7.84^c^	25.03 ± 8.74^d^	370.072	<0.001
Social motivation	5.53 ± 3.29^a^	13.53 ± 4.92^b^	10.15 ± 4.16^c^	12.46 ± 4.44^d^	209.083	<0.001
Autistic mannerisms	4.49 ± 3.76^a^	17.22 ± 6.02^b^	10.97 ± 4.72^c^	12.38 ± 5.76^d^	341.094	<0.001
Total scale	37.98 ± 15.77^a^	91.95 ± 22.90^b^	64.37 ± 19.27^c^	76.87 ± 21.05^d^	401.536	<0.001

Note: values in the same row with the different letter are statistically different at *P* <0.001 (two-tailed)

### Factor analysis

The exploratory factor analysis (EFA) indicated a single underlying factor which accounted for 17.56% of the variance. Of the remaining factors, one accounted for 7.38% of the variance, and the other 18 factors with an eigenvalue of 1 or more, accounted for no more than 4% each, which is typical for a single dimension solution. 47 of the 65 items had factor loadings greater than 0.2 onto factor 1.

By using the data of the ASD group, the factor model was established on the basis of theoretical construct of five factors on the original scale. Parameter estimation was based on a robust assumption of maximum likelihood estimation on the structural equation model. Several model-fitting indices were considered in order to indicate good structural parameters: the Chi-Square (*χ* ^2^), the goodness of fit index (GFI), the adjusted goodness of fit index (AGFI), the non-normed fit index (NFI), the comparative fit index (CFI), and the root mean square error of approximation (RMSEA) which measures the discrepancy between the approximation and the population. Fit indices for the five-factor model of the Chinese SRS base on Confirmatory factor analysis (CFA) using the ASD sample were conducted, with results displaying Chi-square (4055.958, *P* < 0.001), *χ* ^2^/*df* (2.023), GFI (0.566), AGFI (0.536), NFI (0.334), CFI (0.488) and RMSEA (0.072). Good-fit was indicated by non-significant Chi-square (or *χ* ^2^/*df* less than 3), GFI, AGFI, NFI and CFI of more than 0.9 respectively, and RMSEA of less than 0.07 [34], thus it can be expected that this five-factor model fitting is unsatisfactory.

### ROC

**Fig. 4:**
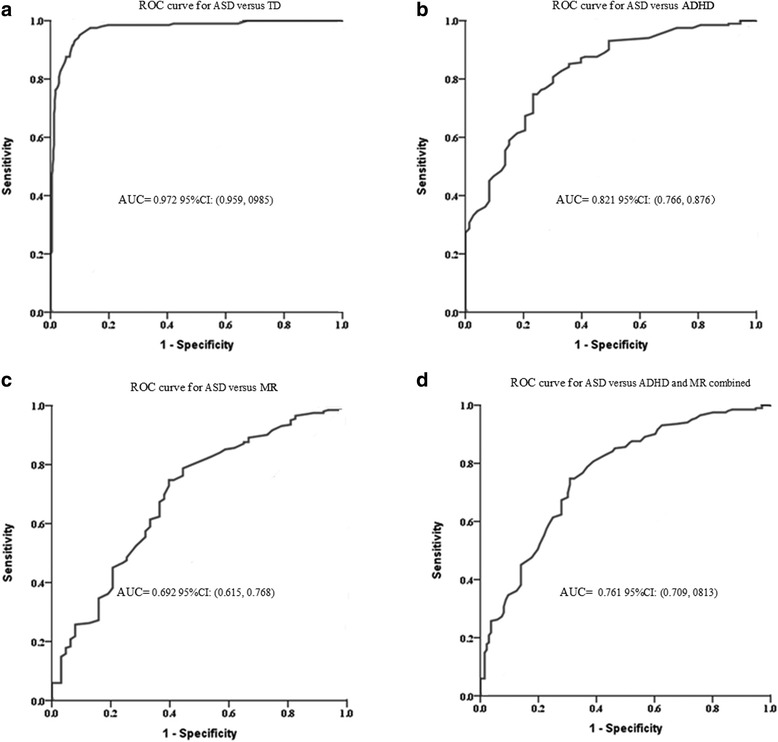
**a** Receiver operator curve of ASD versus TD. **b** Receiver operator curve of ASD versus ADHD. **c** Receiver operator curve of ASD versus MR. **d** Receiver operator curve of ASD versus ADHD and MR combined

The diagnostic validity (value for diagnostic classification) was analyzed by ROC-analyses for ASD vs. TD, ASD vs. ADHD, and ASD vs. MR, as well as for ASD vs. ADHD and MR combined. The ROC curve plotted for the SRS total raw scores (seen in Fig. 4a, b, c, and d) determined the cutoff score on SRS that maximized both sensitivity and specificity, based on the Youden’s index. Table 7 displayed the corresponding AUC, sensitivity, specificity, false-negative rate, false-positive rate, positive-predictive value and negative-predictive value for each measure. The AUC indicates the ability of the tests to correctly classify the individuals with and without an ASD. In this present study, the SRS was predicted to accurately classify 69.2–97.2% of youth ASD correctly.

**Table 7:** Cut-off score, sensitivity, specificity, AUC, FNR, FEP, PPV, NPV based on ROC curve analysis to discriminate ASD and TD control, and ASD and non-ASD clinical groups

	Cut-off	Sensitivity	Specificity	AUC	FNR	FPR	PPV	NPV
ASD vs. TD	56.5	0.950	0.900	0.972*	0.050	0.100	0.824	0.974
ASD vs. ADHD	77.5	0.748	0.767	0.821*	0.252	0.233	0.899	0.523
ASD vs. MR	77.5	0.748	0.603	0.692*	0.252	0.397	0.858	0.427
ASD vs. ADHD and MR combined	77.5	0.691	0.748	0.761*	0.309	0.252	0.782	0.648

Note: *AUC* area under the curve, *FNR* false-negative rate, *FPR* false-positive rate, *PPV* positive-predictive value, *NPV* negative-predictive value* *P* < 0.001

## Discussion

### SRS total raw score means by gender and age

In the U.S normalization data [15], a strong gender effect was found, with males generally rated about 9 SRS Total score points higher than males. Consequently, the differences of the magnitude evidenced in the SRS scores by gender were considered as factors that must be accounted for in norms. In keeping with the U.S. findings, in the UK normalization data, there was significant gender differences with males scoring 4.30 points higher than females. Boys had a 4.30 points higher SRS total raw score than girls of the same age [16]. However, in our study, the mean SRS total scores were 2.88 points higher in boys than in girls among TD group, whereas an opposite pattern was found in the ASD group (2.99 points higher in girls than in boys), but both were not significantly different (*P* > 0.05). These patterns were consistent with that found in the Mexico study [19] in which no significant main effect for gender was found with 3.40 points higher in boys than in girls among control group, whereas an opposite pattern was found (11.3 points higher in girls than in boys) among PDD participants. Similarly, mild gender differences in the normative sample were also reported in the German study [17], which found that the boys were 2.50 points higher than girls in mother ratings and 1.4 points higher than girls in father ratings for the SRS total scores. The author explained that the sex differences are reported here for the sake of establishing norms, but subsequent analyses on the instrument’s psychometric properties were not conducted separately for boys and girls, since the gender differences were relatively modest [17]. Thus, it can be seen, that the magnitude of gender difference for the SRS total score may vary in different samples or different data sources. However, there seemed to be such a trend in all the studies that boys always scored higher than girls on the SRS Total score in the normative sample which is the opposite of what in the clinical sample with girls always scoring higher than boys on the SRS total score, despite of different significant levels. Our study, consistent with Germany [17] and Mexico [19] studies, found that the sex differences of SRS Total score were less pronounced than that in the US normative sample. In this regard, it could be particularly of importance that a substantial part of the U.S.-standardization sample consisted of twins [17]. Research on autism in general and autism traits on the SRS has shown that autism scores in twins, especially males, could be higher than those in the general populations [35, 36].

Our study found no significant differences between preschool-aged and school-aged samples on the SRS total scores either in the TD group, or in any of the clinical subgroups. Furthermore, there was no evidence that would lead to consider a linear correlation with significant level between the SRS total raw score and age in the total sample. This was in line with the U.S. study [15] which found that all of the various age groups were within around 0.2 standard deviation (SD) of the overall group mean, and the correlation of age with the SRS total scores across the 1636 participants resulted in a non-significant coefficient (*r* = 0.02). Similarly, German study demonstrated [17] no noteworthy correlations between age and the SRS total scores in the normative or clinical sample (*r* = −0.06 and 0.00). These findings consistently suggested few age effects on the SRS total raw score in cross-sectional studies.

### Tests-retest reliability and internal consistency

The current study found the SRS tests-retest reliability to be ideal, which replicated the previous findings [15–17, 19, 23], confirming the stability of ratings over time for the SRS. This stability means that large changes in the SRS can be useful for detecting intervention effects. In keeping with the data from the U.S. original [15], Germany [17, 18], the UK [16], Taiwan [23, 24], Mexico [19] and Canada [20], the internal consistency for the SRS total scale of the current study was found excellent, with alpha coefficient in the order of 0.90. However, in our study, two of the five subscales - social awareness and social cognition - were unsatisfactory, with Alpha coefficient lower than 0.70. In addition, we revealed a pattern that the alpha values on “social awareness” (0.277–0.428) were always the lowest, while those on “social communication” (0.726–0.819) were contrarily, the largest in the five subscales, independently of which group or sample was examined. This was consistent with the findings of the previous studies conducted in the UK [16], Mexico [19], Taiwan [23] and Canada [20]. Fombonne et al. [19] have argued that a consistent tendency for alpha is lower for the Awareness subscale and higher for the Communication subscale, most probably reflecting differences in the number of items comprised in each subscale (8 for Awareness and 22 for Communication).

### Concurrent validity

Quite a few previous studies have established the concurrent validity of the SRS with the so-called gold standard clinical ASD instruments, including ADI-R [10, 17, 18, 37–39] and ADOS [17, 18, 38, 40], and with other rapid screening instruments including SCQ [17, 18, 24, 38, 39], CASD [37], SCDC [18], RBQ2 [16], and AQ [41]. The vast majority of these studies have shown moderate to high correlations between the SRS score and the above mentioned instruments’ scores. For example, the study conducted by Bölte et al. [18] revealed correlations with the ADI-R, ADOS, SCQ and SCDC were 0.31–0.45, 0.32–0.35, 0.50, and 0.49, respectively. Taiwan data [24] indicated correlations with SCQ ranging from 0.609 to 0.865. And the SRS U.S. original data [10] demonstrated the SRS total score correlated with the ADI-R algorithm scores with *r* = .0.52–0.79. In this current study, our data revealed that the SRS had an acceptable concurrent validity with the ABC. It can thus be said that the SRS has a preferable and extensive concurrent validation in general.

### Discriminant validity

As expected, we observed the highest SRS total scores in children with ASD, followed by other clinical subgroups and the TD. These data were in keeping with previous studies which all revealed that the ASD group scored dramatically higher than the non-ASD clinical group. Therefore, our data confirmed the previous research findings that the SRS could be used to distinguish the ASD from non-ASD clinical samples. Also, in our current study, despite the fact that ADHD and MR groups scored lower than the ASD group, they still scored remarkably higher than the TD group, which was similar to the previous study findings. For example, in the U.S. standardization sample, the ADHD, mood disorder and other psychiatric disorders were shown to yield intermediate scores, and Taiwan [23] and Germany [17] data revealed similar results. The former revealed that while significantly lower than the average SRS scores in children with ASD, the mean SRS scores of children in the ADHD/developmental delay (DD), ADHD alone, and DD alone groups were still notably higher than those of typical controls. And the latter study showed ADHD, neurotic/emotional disorder, ADHD + CD, CD and other psychiatric disorders to have notably higher mean SRS scores than the TD group. Additionally, as indicated by the study conducted by Pine et al. [42], when scores on the SRS, SCQ and CCC were compared for youths with mood or anxiety disorders, participants with mood and anxiety disorders obtained significantly higher scores on ASD symptom scales. There are several possible explanations for this score pattern. First, screening instruments including SRS may over-identify children at risk of an ASD [42]. Subsequent studies, which included the use of the SRS as a measure of ASD symptomatology, have raised further important considerations regarding the use of this tool in some clinical settings [22]. Therefore, it is important not to over-interpret parent report data alone as indicating “missed diagnosis” [43]. In the ASD assessment and diagnostic clinical setting, this type of “missed diagnosis” or “over identifying” could be avoided by establishing a diagnosis which integrates diverse information or data, including clinical judgment. Secondly, quite a few studies have suggested that varying degrees of ASD traits extend in the general population from healthy individuals to clinical group [29, 44, 45]. ASD may represent the upper extreme of a constellation of traits that are continuously distributed in the population [30]. Hence, the distribution of the intermediate elevated SRS score in the non-ASD clinical sample supported the dimensional perspective of ASD traits. Finally, despite the diagnostic rules applied in ICD-10 and DSM-IV, which do not allow for a co-morbid diagnosis of ASD and ADHD, many research studies have documented the coexistence of ASD and ADHD diagnoses [46]. For example, one recent study reported that 18% of kids with ADHD showed an autism trait profile compared to 0.87% of the controls [47]. Another recent study indicated that nearly one third of children with ASD also showed clinically significant symptoms of ADHD [48]. Consequently, our study replicated the previous research findings of substantial overlap of symptomatology between ASD and ADHD, and highlights the importance of specifically examining the co-existing autistic traits in children with ADHD for better characterization of the underlying physiopathology and treatment [49]. On this occasion, the SRS can help to obtain a more comprehensive understanding of these individuals’ social skills deficits.

### Construct validity

In our study, the goodness-of-fit indices from the CFA indicated a poor fit for the five-factor structure comprising all 65 items of the SRS, which was consistent with the findings of the Canadian study [20], in which the authors also found that the two other tested models, a 5-factor first-order model and a 5-factor second-order model, with the subscales as factors, did not fit well. However, a study from Taiwan [24] found that the CFA of the normative sample had revealed a 4-factor model to fit well, which was demonstrated primarily by the EFA after excluding five items with low correlation coefficients. Thus, the five theoretic dimensions of the SRS could not be confirmed by the existing studies. Nevertheless, it seems reasonable, because the clustering of the original five subscales was based on clinical experience and use of intervention strategies, rather than by a confirmation through factor analysis [29]. As recommend by the developers, the five theoretical subscales should only be used for the purpose of clinical description, including detection of the intervention effects.

The EFA in the ASD sample of our present study indicated a single underlying factor which accounted for 17.56% of the variance, and all subsequent factors explained markedly less variance than the first. This result was consistent with previous studies that supported a single-factor solution with varying degree of variance in different samples, e.g. the U.S normative study yielded a single-factor structure in both the normative and clinical samples, with the first factor accounting for the variance up to 70% in school sample [15]. In the German data [17], 34.9% of variance in the clinical sample, and 16.5% (father rating), 17.5% (mother rating) of variance in the normative sample were explained as variance by the first factor. In addition, the UK [16] normative sample had the first factor accounting for 19% of the variance, which was similar normative sample from Germany [17]. With respect to the differences in magnitude of variance explaining for the one-factor structure, Bölte et al. [17] suggest that it may have occurred on the basis of differences in age of the subjects in the respective samples, and the fact that in the published American school samples, informants were teachers (rather than parents), who each provided ratings on more than one student (15 on average). Another possible reason may be the different methods applied in the EFA. Generally, it is reasonable to regard the aforementioned findings as supporting a one-factor structure, and the SRS yield an overall autism score that is consistent with the notion of a single spectrum. At the same time, this result implied the total score rather than the subscale score or individual subscales score combination should be applied when using SRS for assist making decision of ASD.

In addition, the Social Responsiveness Scale has been revised to the Social Responsiveness Scale-2 [50]. In the new assessment, the former five factors have been revised to be identified as "treatment clusters." A two-factor structure (corresponding to social communication impairment and restricted, repetitive behavior) as elaborated in the updated Diagnostic and Statistical Manual of Mental Disorders (5th ed.; DSM-5) criteria for autism spectrum disorder exhibited acceptable model fit in confirmatory factor analysis [51]. This implies that the scoring of broad autism traits in the SRS-2 may be helpful in diagnostic contexts where separate measurements of DSM-5 domains are desired. Therefore, future studies are worthy to use our present data from the SRS and revise the analysis to use the SRS-2 norms and factors.

### ROC

In ROC analyses, in keeping with the results of data from Germany (AUC =0.98) [18], Mexico (AUC =0.962) [19], Taiwan (AUC =0.997) [23], US (AUC =0.988) [38], and Germany (AUC =1.0) [39], the current study showed the parent-rating SRS total score to be distinguished excellently between ASD and TD (AUC = 0.972). In clinical practice, the SRS is generally used to indicate children for further diagnostic evaluation, thus requiring high sensitivity. Our study shows that the total raw cut-off score of 56.5 with a sensitivity of 0.950 could serve this purpose very well. Moreover, the aim to identify as much ASD cases as possible will go at the expense of the specificity, i.e. a considerable proportion of non-ASD cases will also be identified and selected for further diagnostic assessment, with the accompanying costs and burden on the families. In our study, the total raw cut-off score of 56.5 with the specificity of 0.90 is also effective in correctly identifying children who do not need further ASD-specific diagnostic assessment.

We also applied the SRS approach for discriminate the ASD from the non-ASD clinical subgroups. The AUC was significantly lower, with 0.821 for ASD vs. ADHD and 0.761 for ASD vs. non-ASD clinical combined, respectively. The sensitivities were 0.748/0.691, and specificities were 0.767/0.748, respectively, for the ASD using 77.5 as the cut-off SRS score. This was in line with the previous studies in which ASD vs. ADHD (AUC =0.86 ~ 0.88) [17, 18, 23], ASD vs. ODD/CD (AUC =0.82) [39], ASD vs. ADHD and other non-ASD clinical combined (AUC =0.81 ~ 0.879) [15, 17, 18, 23] were compared. However, the present studies so far may be inappropriate for a solid clinical classification due to the insufficient sensitivity and specificity. Moreover, the use of separate cut-off score of the SRS for screening purposes (optimizing sensitivity) vs. clinical confirmation (optimizing specificity), are well worth considering, as discussed in the SRS Manual [17]. Choosing a higher cut-off point may be preferable when it is important to further minimize the number of false positives, i.e. children with non-ASD who are incorrectly identified at risk for ASD. Thus, clinicians and researchers should be aware of the trade-off between maximizing the identification of children at risk versus minimizing the number of children targeted to receive further assessments, when selecting the cut-off that best serves their particular purpose or the population screened.

While the findings from this current study provide some guidance regarding the clinical utility of this screening instrument, there are several limitations that need to be acknowledged. First of all, our sample was from Guangzhou, so our results should not be generalized to the whole population in Mainland China. Further population-based studies are needed to clarify the utility of the SRS in more diverse samples. Secondly, the ASD diagnoses of our study were not confirmed using standardized assessments including ADI-R and ADOS, because these assessment instruments for the Chinese version had not been available for the utilization in Mainland China. This may have influenced the SRS score distribution of the ASD subgroup. Thus, it is possible that some of the discrepancies observed between the SRS score and the ASD diagnosis were due to variability in how children were assessed and diagnosed in the clinical setting [23]. All the clinical diagnoses in our study were established by extensively- experienced developmental and behavioral pediatricians from a medical center well-known for its specializing in the services for these conditions. Therefore, this concern should not have had a significant impact on the study’s findings. However, further studies involving the ASD sample confirmed by gold standard instruments, including ADI-R and ADOS, should be warranted to further clarify the usefulness of the SRS. Thirdly, regarding the concurrent validity, only one ASD screening tool, i.e. the ABC, was used to examine the correlation with the SRS in our study. It may have been insufficient to demonstrate this validity. Thus, further studies are needed to investigate this type of validity involving diverse ASD screening tools and ASD diagnostic assessments, particularly the ADI-R and ADOS, which are viewed as the “gold standard” for diagnosing the ASD. Fourthly, our study is also limited by it’s lack of the inter-tester reliability measure. Respondents in our study were completely parents (for the most part, mothers), and subsequent analyses of the instrument’s psychometric properties were not conducted separately for mothers and fathers. Therefore, it may have had an influence on the SRS score results, given that the U.S. normative study [15] as shown a difference between maternal, paternal, and teacher rating. As in previous research, moderately high correlations were reported between parent and teacher SRS rating and between father and mother SRS rating. For example, high correlations have been reported in the validation U.S. studies [15, 40], with parent-teacher correlations in the range of 0.70 and over, and, the studies conducted by Bölte et al. [17] and Pearl et al. [52], both demonstrating excellent father-mother SRS rating correlations with r =0.76–0.95. However, the study by Fombonne et al. [19] reported only a mild to intermediate correlation between parent and teacher SRS rating, with *r* =0.22–0.50. Besides, it should be noted that the correlation between informants was much higher in the clinical sample as compared to the control group [19]. As a result, further studies should be warranted to systematically investigate the SRS score distributions in different respondents, and discrepancies between different respondents, particularly in the teacher respondents. It is because once the children attend schools, including kindergarten and primary schools, the teachers play a very important role as observers in the neutral context. They are able to gather information from a multidisciplinary team work [23], which is necessary and helpful for an accurate assessment of SRS used, as we know, primarily to evaluate the individual’s reciprocal social behavior.

## Conclusions

In summary, the current study extends our knowledge regarding the clinical utility of the Chinese version of the SRS in the sample from Mainland China. Internal consistency, test–retest reliability, convergent validity and discriminant validity of our data were satisfactory to good. This further confirms that the SRS is an excellent method for identifying ASD classification with a high sensitivity and specificity; however, when applied for distinguishing the ASD from the non-ASD clinical subgroup, the sensitivity and specificity were lower. The results of the exploratory factor analysis were consistent with those as reported for the SRS original in indicating a single-factor structure in clinical samples. Our findings support the SRS as a parent-report measure of quantitative autistic impairment in the Mainland China. But, the use of separate cut-offs for screening purposes (optimizing sensitivity) vs. clinical confirmation (optimizing specificity) should be considered.
